# Deep Learning Model for Predicting the Pathological Complete Response to Neoadjuvant Chemoradiotherapy of Locally Advanced Rectal Cancer

**DOI:** 10.3389/fonc.2022.807264

**Published:** 2022-06-08

**Authors:** Xiaoying Lou, Niyun Zhou, Lili Feng, Zhenhui Li, Yuqi Fang, Xinjuan Fan, Yihong Ling, Hailing Liu, Xuan Zou, Jing Wang, Junzhou Huang, Jingping Yun, Jianhua Yao, Yan Huang

**Affiliations:** ^1^ Department of Pathology, The Sixth Affiliated Hospital of Sun Yat-sen University, Guangzhou, China; ^2^ Guangdong Institute of Gastroenterology, The Sixth Affiliated Hospital of Sun Yat-sen University, Guangzhou, China; ^3^ Tencent AI Lab, Shenzhen, China; ^4^ Department of Radiation Oncology, The Sixth Affiliated Hospital of Sun Yat-sen University, Guangzhou, China; ^5^ Department of Pathology, Yunnan Cancer Hospital, Kunming, China; ^6^ Department of Electronic Engineering, The Chinese University of Hong Kong, Hong Kong, Hong Kong SAR, China; ^7^ Department of Pathology, Cancer Center of Sun Yat-sen University, Guangzhou, China

**Keywords:** rectal cancer, deep learning, neoadjuvant chemoradiotherapy, pathological complete response, artificial intelligence

## Abstract

**Objective:**

This study aimed to develop an artificial intelligence model for predicting the pathological complete response (pCR) to neoadjuvant chemoradiotherapy (nCRT) of locally advanced rectal cancer (LARC) using digital pathological images.

**Background:**

nCRT followed by total mesorectal excision (TME) is a standard treatment strategy for patients with LARC. Predicting the PCR to nCRT of LARC remine difficulty.

**Methods:**

842 LARC patients treated with standard nCRT from three medical centers were retrospectively recruited and subgrouped into the training, testing and external validation sets. Treatment response was classified as pCR and non-pCR based on the pathological diagnosis after surgery as the ground truth. The hematoxylin & eosin (H&E)-stained biopsy slides were manually annotated and used to develop a deep pathological complete response (DeepPCR) prediction model by deep learning.

**Results:**

The proposed DeepPCR model achieved an AUC-ROC of 0.710 (95% CI: 0.595, 0.808) in the testing cohort. Similarly, in the external validation cohort, the DeepPCR model achieved an AUC-ROC of 0.723 (95% CI: 0.591, 0.844). The sensitivity and specificity of the DeepPCR model were 72.6% and 46.9% in the testing set and 72.5% and 62.7% in the external validation cohort, respectively. Multivariate logistic regression analysis showed that the DeepPCR model was an independent predictive factor of nCRT (*P*=0.008 and *P*=0.004 for the testing set and external validation set, respectively).

**Conclusions:**

The DeepPCR model showed high accuracy in predicting pCR and served as an independent predictive factor for pCR. The model can be used to assist in clinical treatment decision making before surgery.

## Introduction

Colorectal cancer remains one of the leading causes of cancer death (1). For patients with locally advanced rectal cancer (LARC), neoadjuvant chemoradiotherapy (nCRT) followed by total mesorectal excision (TME) is recommended as a standard treatment strategy. nCRT can significantly reduce local recurrence and treatment-associated toxicity and more importantly, make tumors more amenable to resection. However, the treatment response to nCRT varies greatly among patients. Approximately 15-38% of patients could obtain a pathological complete response (pCR) and are recommended the watch and wait approach to avoid the side effects of surgery (2), while 20% of patients have little to no response to nCRT and might even suffer significant side effects and miss their best opportunity for surgery (3–5). More importantly, patients with pCR have better long-term outcomes, indicating a favorable prognosis (6). However, how to predict treatment response, especially to identify pCR candidates prior to nCRT, remains challenging for LARC.

Previous studies have shown that tumor stage, serum tumor markers before neoadjuvant therapy, and lymphocyte infiltration in the tumor microenvironment are associated with tumor regression to nCRT (7). Recently, with the development of artificial intelligence algorithms, radiological imaging has been used to evaluate the treatment response of LARC (8–14). The commonly adopted imaging techniques include diffusion-weighted magnetic resonance imaging (MRI) (11), diffusion kurtosis and T2-weighted MRI (8), and a multiparametric MRI protocol with dynamic-contrast-enhanced MRI (13). For instance, Zhang *et al.* (10) developed a pCR prediction model based on diffusion kurtosis and T2-weighted MRI, and the area under the curve (AUC) was 0.70 (95% confidence interval (CI): 0.59, 0.79). Histopathological images prevail as the gold standard for patient diagnosis and contain abundant biological information. Therefore, we anticipate that more accurate predictions can be achieved by analyzing pathological images than by analyzing radiological images.

Compared with conventional machine learning, deep learning can automatically extract features from an image without the necessity of feature predefinition and is suitable for mining the most relevant feature representations. Multi-instance learning (MIL), as a weakly supervised deep learning technique, has achieved promising results on the topic of patient prognosis and outcome prediction (15–18). MIL enables the network to learn more holistic information from whole-slide images (WSIs). To the best of our knowledge, there has been little investigation on the prediction of pCR based on histopathological images prior to nCRT with the MIL technique. The aim of this study was to develop a deep pathological complete response (DeepPCR) prediction model for the prediction of pCR directly from conventional hematoxylin & eosin (H&E)-stained histopathological images.

## Materials and Methods

### Study Cohort and Availability

Two different cohorts, i.e., the primary cohort and external validation cohort, were adopted for training and internal and external validation and included retrospectively identified LARC patients from January 1, 2010, to January 1, 2018, from three hospitals in China (the Sixth Affiliated Hospital of Sun Yat-sen University, Cancer Center of Sun Yat-sen University, and Yunnan Cancer Hospital). A total of 842 patients were recruited; among them, the primary cohort (783 patients from the Sixth Affiliated Hospital of Sun Yat-sen University and Cancer Center of Sun Yat-sen University) was randomly subgrouped into the training set (666 patients, 85%) and testing set (117 patients, 15%), and the external validation cohort (from Yunnan Cancer Hospital) contained 102 patients. The inclusion criteria were as follows: (1) patients had locally advanced disease determined by pretreatment TNM stage (T3/T4, and/or N+); (2) biopsy was performed, and the biopsy specimen was pathologically diagnosed as adenocarcinoma; and (3) patients underwent nCRT followed by rectal resection. The exclusion criteria were as follows: (1) patients with familial adenomatous polyposis, distant metastases, or Lynch syndrome; and (2) patients with no information on tumor regression grade (TRG) and no available H&E-stained slides.

**Figure 1:**
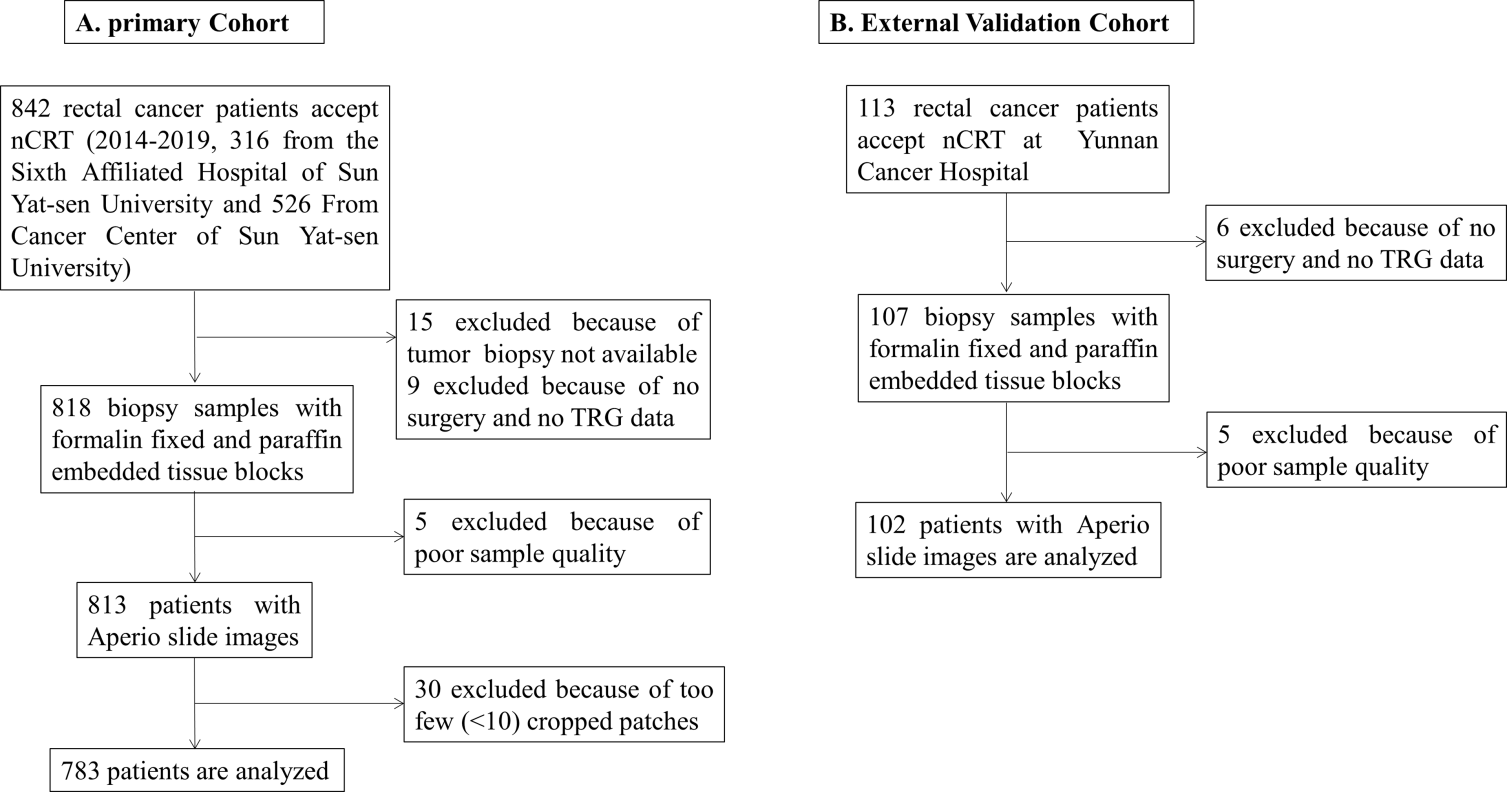
The flow diagram of patient enrollment. **(A)** Primary cohort, **(B)** External validation cohort.

All patients accepted a standard treatment strategy based on the National Comprehensive Cancer Network (NCCN) guidelines (version 3, 2017). The nCRT regimen was 50 Gy pelvic radiation therapy with concurrent 5-fluorouracil-based chemotherapy (FOLFIRI or FOLFOX regimens). TME was performed by either anterior resection or abdominoperineal resection after nCRT of 4-8 weeks. The TRG after nCRT was used to divide patients into two groups based on H&E-stained slides after surgery: pCR (with no remaining viable cancer cells) and non-pCR (with small clusters of cancer cells or no response with extensive residual cancer). The flow diagram of patient enrollment into the two cohorts is shown in **Figure 1**.

Clinicopathological variables, such as age, sex, TNM stage, histological grade, TRG after surgery, and blood testing parameters, including lymphocytes, neutrophils, carcinoembryonic antigen (CEA), carbohydrate antigen 19-9 (CA19-9), and lactate dehydrogenase (LDH) prior to nCRT treatment, were collected. This study was approved by the Institutional Review Board of the Sixth Affiliated Hospital of Sun Yat-sen University.

### Data Preparation

Formalin-fixed paraffin-embedded (FFPE) biopsy tissue blocks were cut into 4-µm sections for H&E staining. All slides were checked by a pathologist who ascertained that they contained tumor areas. WSIs were acquired at a magnification of 20× on an Aperio scanner.

Tumor tissue regions were hand-delineated by pathologists (Dr. XYL and Dr. HLL) using Aperio Image Scope software and subsequently cropped into patches with a size of 299×299 pixels at a magnification of 20×. The distribution of the number of patches per slide followed a long-tail distribution, with the majority of slides containing approximately 100 patches. For slides with more than 1000 patches, we randomly chose 1000 cropped patches.

### pCR Candidate Classification

**Figure 2:**
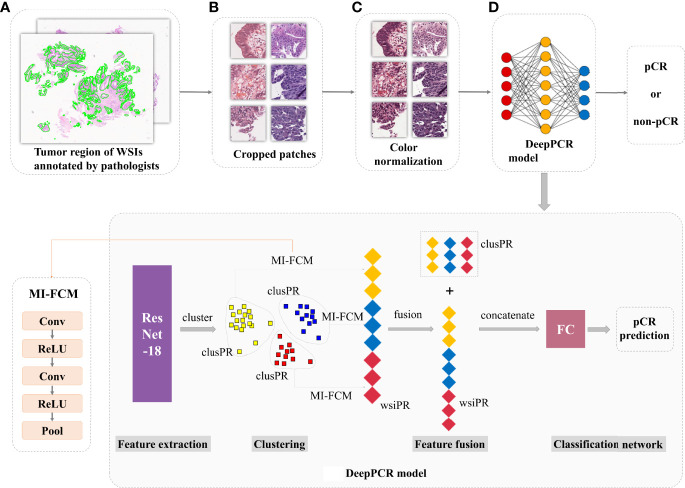
The proposed deep learning framework (DeepPCR) for pCR prediction. **(A)** WSIs with tumors annotated by expert pathologists. **(B)** All WSIs were cropped into small patches with a size of 299×299 pixels at a magnification of 20×. **(C)** An in-house deep learning-based color normalization method was applied to ensure the color consistency of the cropped patches. **(D)** Illustration of the proposed DeepPCR model for pCR candidate prediction. Three scales of phenotype feature representations (i.e., patchPR, clusPR, and wsiPR) were integrated to derive the final prediction.

Four models were designed for classifying the input biopsy histological images, with patients’ distinct TRG outcomes as the ground truth. The first three models were trained on 102,728 patches and tested on 18475 patches in the primary cohort, namely, the DeepPCR model, *patch-based combined* model, and *patch-based individual model.* The DeepPCR model was built upon the MIL strategy (**Figure 2**). Specifically, a pretrained ResNet-18 model (19) was leveraged to extract the pathological feature representations of each cropped patch, i.e., the patch-wise phenotype representation (patchPR). Based on the patchPRs, the unsupervised K-means algorithm was used to categorize these features into six clusters (see **Supplementary Figure 1**). Each cluster occupied a subspace of the features and comprised a distinctive phenotype group. The patches in each cluster were further processed by a multi-instance fully convolutional model (MI-FCM) (20) to generate cluster-wise phenotype representation (clusPR). Herein, the MI-FCM was comprised of two pairs of *Conv-ReLU* layers, followed by a *pooling* layer. Afterwards, WSI-wise phenotype representation (wsiPR) was constructed by concatenating the clusPRs from the same WSI. The wsiPR sufficiently exploited the intercluster feature difference and intracluster feature dependence, constituting the most informative phenotype representation. Based on wsiPR, a two-layer fully connected network was leveraged to generate the final prediction. The DeepPCR model built a hierarchical feature structure from patch to WSI and explicitly modeled the mutual dependence between different phenotype groups for patient outcome prediction.

*The patch-based combined model* and *patch-based individual model* used patch-based approaches in which the cropped patches shared the same label with the original histopathological WSI and the prediction of patch-based methods was made for each patch rather than each WSI. Similar to DeepPCR, the pretrained ResNet-18 model was adopted to extract the phenotype representations of each cropped patch. According to the aggregation method of the patch-level prediction, we implemented these patch-based models in two ways. One was to predict each individual patch’s label, and then combined them *via* majority voting, which was called the *patch-based individual model*. The other was to aggregate the patch-level predictions of each subject by removing the clustering step in DeepPCR, called the *patch-based combined model* (remaining modules are the same as DeepPCR). To validate the effectiveness of pathological imaging data in pCR outcome prediction compared with nonpathological data, the fourth model (*hematology model*), based on clinical hematology data, including CEA, CA19-9, LDH, lymphocytes, and neutrophils, was built. A two-layer multilayer perceptron (MLP) model was adopted in the hematology model.

### Phenotype Visualization

To visualize the representative phenotypes in each K-means cluster, t-distributed Stochastic Neighbor Embedding (t-SNE) (21) and the Raster Fairy method (22) were applied on the patchPRs. t-SNE is a technique for dimensionality reduction that is particularly well suited for the visualization of high-dimensional data. The Raster Fairy method aims to transform the two-dimensional clustering data derived from t-SNE into a regular grid without destroying the neighborhood relations emerging from the clustering. The GradCAM method (23) was used to calculate the patch importance for target prediction.

### Statistical Analysis

The predictive efficacy of the model was evaluated by the area under the receiver operating characteristic curve (AUC-ROC), area under the precision-recall curve (AUC-PR), sensitivity, specificity, positive predictive value (PPV), and negative predictive value (NPV). Univariate and multivariate logistic regression analyses were performed to investigate the predictive value for all biomarkers. The statistical significance of the differences in the clinicopathological characteristics of pCR and non-pCR patients were calculated using the Mann-Whitney test (two-tailed) for continuous variables and Fisher’s exact test (two-tailed) for dichotomous variables. Comparisons of clinicopathological factors in the primary and external validation cohorts were performed using Student’s t test for continuous variables and Fisher’s exact test (two-tailed) for dichotomous variables. A two-sided p value of less than 0.05 was considered statistically significant.

## Results

### Patient Characteristics

The primary cohort included 783 patients: 295 patients from the Sixth Affiliated Hospital of Sun Yat-sen University and 488 patients from the Cancer Center of Sun Yat-sen University. A total of 201 and 582 patients were classified as pCR and non-pCR, respectively. The external validation cohort from Yunnan Cancer Hospital included 102 patients, of which 24 and 78 patients were classified as pCR and non-pCR, respectively. The clinicopathological characteristics of the patients in the primary and external validation cohorts are provided in **Table 1**. The clinicopathological characteristics, including clinical T stage and histological grade, were different between the primary and external validation cohorts (P<0.001 and P<0.001, respectively) (**Supplementary Table 1**).

**Table 1:** Clinicopathological characteristics of patients in the training, testing, and external validation cohorts.

	Training set (n=666)			Testing set (n=117)			ExternalValidation set (n=102)		
	PCR (%)(n=171)	Non-PCR (%)(n=495)	*P* value	PCR (%)(n=30)	Non-PCR (%)(n=87)	*P* value	PCR (%)(n=24)	Non-PCR (%)(n=78)	*P* value
**Age, mean(SD), y**	52.77 ± 12.02	54.71 ± 11.78	0.078	53.90 ± 11.71	55.38 ± 11.47	0.549	54.08 ± 11.01	57.17 ± 10.37	0.182
**Sex, No. (%)**			0.849			0.376			0.081
**Female**	55(32.2)	154(31.1)		8(26.7)	32(36.8)		12(50.0)	22(28.2)	
**Male**	116(67.8)	341(68.9)		22(73.3)	55(63.2)		12(50.0)	56(71.8)	
**Clinical T stage**									
**cT2**	10(5.9)	16(3.2)	0.124	2(6.7)	2(2.3)	0.271	1(4.2)	0(0.0)	0.235
**cT3**	113(66.1)	323(65.3)	0.926	20(66.7)	55(63.2)	0.827	9(37.5)	24(30.8)	0.612
**cT4**	48(28.0)	156(31.5)	0.442	8(26.6)	30(34.5)	0.503	14(58.3)	54(69.2)	0.333
**Clinical N stage**									
**cN0**	34(19.9)	76(15.4)	0.189	5(16.7)	13(14.9)	0.777	0(0.0)	18(23.1)	0.006
**cN1**	86(50.3)	249(50.3)	1	13(43.3)	40(46.0)	0.834	17(70.8)	43(55.1)	0.236
**cN2**	51(29.8)	170(34.3)	0.301	12(40.0)	34(39.1)	1	7(29.2)	17(21.8)	0.582
**TNM stage**									
**Stage II**	35(20.5)	76(15.3)	0.167	5(16.6)	13(14.9)	0.777	0(0.0)	18(23.1)	0.006
**Stage III**	136(79.5)	419(84.7)	0.124	25(83.4)	74(85.1)	0.777	24(100.0)	60(76.9)	0.006
**Histological grade**									
**1**	22(12.9)	55(11.1)	0.579	3(10.0)	16(18.4)	0.394	1(4.2)	0(0)	0.235
**2**	125(73.1)	382(77.2)	0.299	22(73.3)	65(74.7)	1	23(95.8)	71(91.0)	0.677
**3**	24(14.0)	58(11.7)	0.421	5(16.7)	6(6.9)	0.147	0(0)	7(9.0)	0.194
**Patch No.**	102,728			18475			46599		

### pCR Candidate Prediction in the Primary Cohort

**Figure 3:**
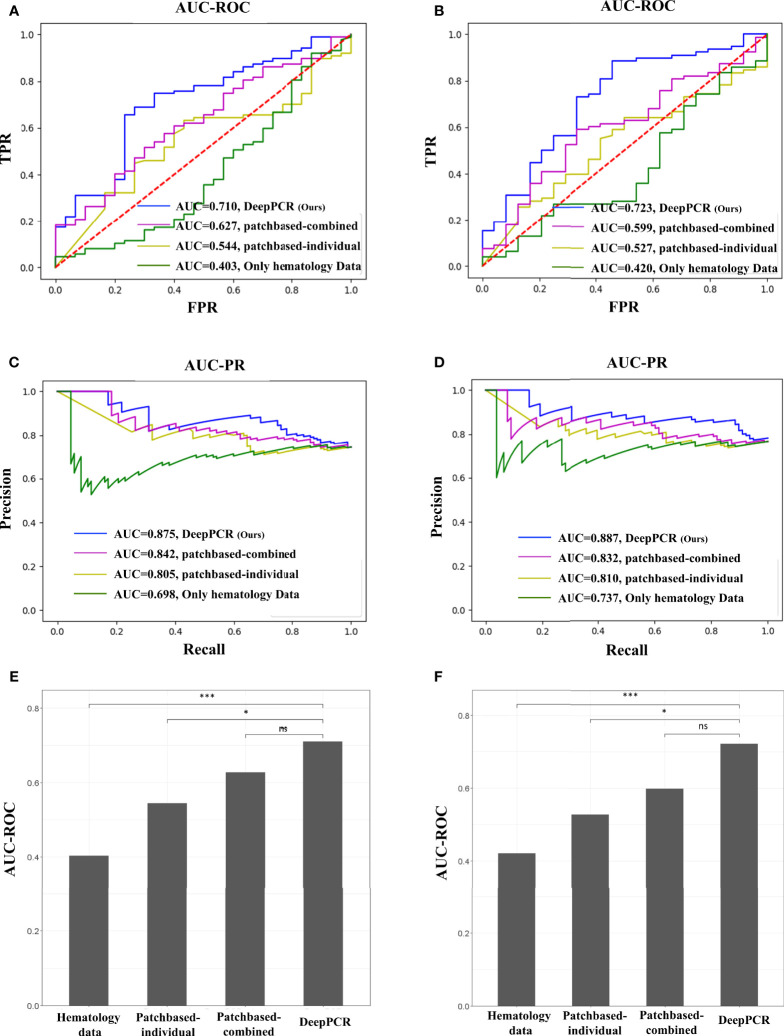
**(A, B)** AUC-ROC of the four comparative methods in the **(A)** primary and **(B)** external validation cohorts (top row). **(C, D)** AUC-PR of the four comparative methods in the **(C)** primary and **(D)** external validation cohorts (middle row). **(E, F)** DeLong test for the four comparative methods in the **(E)** primary and **(F)** external validation cohorts (bottom row). In this work, we used a probability threshold of 0.7 (that is, any patient with a pCR prediction probability greater than 0.7 was reported as a pCR candidate). No significant difference (ns): *P* > 0.05, *P < 0.05, ***P < 0.001.

The DeepPCR model had a higher discriminative power, with an AUC-ROC of 0.710 (95% CI: 0.595, 0.808) and an AUC-PR of 0.875 (95% CI: 0.795, 0.935) in the primary cohort (**Figure 3A** and **Table 2A**). The sensitivity, specificity, PPV and NPV were 72.6%, 46.9%, 70.4%, and 54.0%, respectively (**Table 2A**). The other three models showed inferior performance. Specifically, the *hematology model* had an AUC-ROC of 0.403 (95% CI: 0.274, 0.534) and an AUC-PR of 0.698 (95% CI: 0.591, 0.805). *The patch-based individual* model and *patch-based combined* model achieved an AUC-ROC of 0.544 (95% CI: 0.432, 0.653) and an AUC-PR of 0.805 (95% CI: 0.717, 0.885) and an AUC-ROC of 0.627 (95% CI: 0.516, 0.733) and an AUC-PR of 0.842 (95% CI: 0.762, 0.909), respectively (**Figures 3A, C** and **Table 2A**). As shown in **Figure 3E**, the AUC-ROC of the DeepPCR model was significantly higher than that of the *hematology model (P < 0.001*) and *patch-based individual model (P < *0 .05*).*

**Table 2:** Results of DeepPCR and the comparative models in the (a) primary and (b) external validation cohorts.

(a) Model/Outcome	AUC-ROC	AUC-PR	Sen (%)	Spe (%)	PPV (%)	NPV (%)
**Hematology model**	0.403 (0.274, 0.534)	0.698 (0.591, 0.805)	72.6 (64.1, 80.3)	27.2 (18.8, 36.2)	61.7 (60.0, 71.1)	37.7 (30.8, 51.2)
**Patch-based individual model**	0.544 (0.432, 0.653)	0.805 (0.717, 0.885)	68.4 (59.8, 76.9)	25.8 (17.0, 34.7)	57.2 (45.2, 69.6)	27.0 (15.4, 46.8)
**Patch-based combined model**	0.627 (0.516, 0.733)	0.842 (0.762, 0.909)	69.2 (60.7, 77.8)	30.4 (20.7, 40.7)	61.6 (50.5, 73.1)	37.6 (18.0, 59.4)
**DeepPCR model**	0.710 (0.595, 0.808)	0.875 (0.795, 0.935)	72.6 (64.1, 80.3)	46.9 (32.6, 61.0)	70.4 (61, 79.9)	54.0 (35.8, 70.9)
**(b) Model/Outcome**	**AUC-ROC**	**AUC-PR**	**Sen (%)**	**Spe (%)**	**PPV (%)**	**NPV (%)**
**Hematology model**	0.420 (0.293, 0.548)	0.737 (0.623, 0.846)	70.6 (61.8, 79.4)	21. 7 (14.2, 30.0)	57.4 (48.5, 67.4)	17.6 (14.3, 20.4)
**Patch-based individual model**	0.527 (0.402, 0.657)	0.810 (0.712, 0.895)	73.5 (64.7, 81.4)	22.6 (15.3, 31.4)	57.9 (62.6, 72.4)	17.8 (17.4, 18.1)
**Patch-based combined model**	0.599 (0.474, 0.726)	0.832 (0.732, 0.919)	69.6 (60.8, 78.4)	27.2 (16.3, 38)	62.3 (49.9, 74.5)	31.7 (14.8, 54.1)
**DeepPCR model**	0.723 (0.591, 0.844)	0.887 (0.805, 0.949)	72.5 (63.7, 81.4)	62.7 (46.3, 77.3)	75.8 (67.1, 84.7)	53.6 (36.8, 68.8)

The CI value is inside the parentheses. Sen, sensitivity; Spe, specificity; PPV, positive predictive value; NPV, negative predictive value. In this work, we used a probability threshold of 0.7 (that is, any patient with a pCR prediction probability greater than 0.7 was reported as a pCR candidate).

### pCR Candidate Prediction in the External Validation Cohort

To investigate the effectiveness and generalizability of the DeepPCR model, it was validated in the external cohort. In the external validation cohort, the DeepPCR model achieved a similar AUC-ROC of 0.723 (95% CI: 0.591, 0.844) and an AUC-PR of 0.887 (95% CI: 0.805, 0.949) (**Figures 3B, D** and **Table 2B**). The sensitivity, specificity, PPV and NPV were 0.725 (95% CI: 0.637, 0.814), 0.627 (95% CI: 0.463, 0.773), 0.758 (95% CI: 0.671, 0.847), and 0.536 (95% CI: 0.368, 0.688), respectively (**Table 2B**). In external cohorts, the AUC-ROC of the DeepPCR model was significantly higher than that of the hematology model (P < 0.001) and patch-based individual model (P < 0 .05) (**Figure 3F**).

### Univariate and Multivariate Analyses

In the primary cohort, the univariate logistic regression analysis showed that CEA and DeepPCR model were significantly correlated with pCR (*P=0*.033 and 0.0001, respectively) (**Table 3A**). Multivariate logistic regression analysis showed that only DeepPCR was an independent factor for predicting pCR (95% CI: 1.646, 28.743; *P*=0.008) (**Table 3B**).

**Table 3:** Univariate and multivariate logistic regression analyses.

(a) Univariate logistic regression	Testing Set		External Validation Set	
	P value	Exp (B) (95% CI)	P value	Exp (B) (95% CI)
Sex	0.316	1.6 (0.638, 4.011)	0.051	0.393 (0.153, 1.006)
Age	0.051	2.679 (0.995, 7.212)	0.042	2.768 (1.039, 7.376)
TNM stage	0.822	1.138 (0.369, 3.513)	0.998	0 (0, -)
CEA	0.033	2.796 (1.087, 7.197)	0.029	3.667 (1.145, 11.74)
CA-199	0.087	2.128 (0.896, 5.055)	0.054	2.505 (0.985, 6.37)
CRP	0.198	2.348 (0.639, 8.621)	–	
LDH	0.999	5.80e8 (0, -)	0.207	2.4 (0.617, 9.339)
Lymphocytes	0.24	2.186 (0.593, 8.062)	0.133	2.2 (0.788, 6.146)
Neutrophils	0.414	1.524 (0.555, 4.186)	0.097	2.508 (0.846, 7.436)
NLR	0.142	3.155 (0.681, 14.623)	0.04	3.045 (1.054, 8.804)
Patch-indi	0.06	2.248 (0.967, 5.224)	0.219	1.786 (0.709, 4.5)
Patch-comb	0.053	2.548 (0.989, 6.564)	0.023	3.143 (1.171, 8.437)
DeepPCR	0.0001	6.125 (2.462, 15.239)	0.0001	7 (2.575, 19.028)
**(b) Multivariate logistic regression**	**Test Cohort**		**External Validation Cohort**	
	**Sig.**	**Exp (B) (95% CI)**	**Sig.**	**Exp (B) (95% CI)**
Sex	0.143	2.45 (0.739, 8.124)	0.011	0.122 (0.024, 0.621)
Age	0.489	1.576 (0.434, 5.72)	0.705	1.346 (0.289, 6.261)
TNM stage	0.965	1.034 (0.233, 4.582)	0.998	0 (0, -)
CEA	0.101	2.718 (0.823, 8.973)	0.189	3.211 (0.564, 18.284)
CA-199	0.124	2.413 (0.785, 7.415)	0.059	4.137 (0.945, 18.108)
CRP	0.104	4.607 (0.732, 29.003)		
LDH	0.999	2.6e8 (0, -)	0.118	7.334 (0.604, 89.051)
Lymphocytes	0.128	3.412 (0.704, 16.539)	0.203	3.418 (0.514, 22.723)
Neutrophils	0.979	0.981 (0.239, 4.023)	0.874	0.846 (0.107, 6.699)
NLR	0.138	4.242 (0.628, 28.678)	0.05	8.854 (0.995, 78.749)
Patch-indi	0.346	1.657 (0.58, 4.732)	0.831	0.855 (0.204, 3.591)
Patch-comb	0.8	0.819 (0.175, 3.842)	0.642	1.453 (0.301, 7.023)
DeepPCR	0.008	6.879 (1.646, 28.743)	0.004	10.461 (2.138, 51.186)

(a) Univariate logistic regression analysis of the testing set and external validation set. (b) Multivariate logistic regression analysis of the testing set and external validation set. The covariates were sex, age, TNM stage, CEA, CA19-9, CRP, LDH, lymphocytes, neutrophils, neutrophil-to-lymphocyte ratio (NLR), patch-based individual (patch-indi) model, patch-based combined (patch-comb) model, and DeepPCR model.

In the external validation cohort, age, CEA, neutrophil-to-lymphocyte ratio (NLR), patch-based combined model and DeepPCR model were significantly correlated with pCR (*P=0*.042, 0.029, 0.04, 0.023, and 0.0001, respectively) (**Table 3A**). Multivariate logistic regression analysis showed that only DeepPCR was an independent factor for predicting pCR (95% CI: 2.138, 51.186; *P*=0.004) (**Table 3B**).

### Histological Patterns Associated With TRG

**Figure 4:**
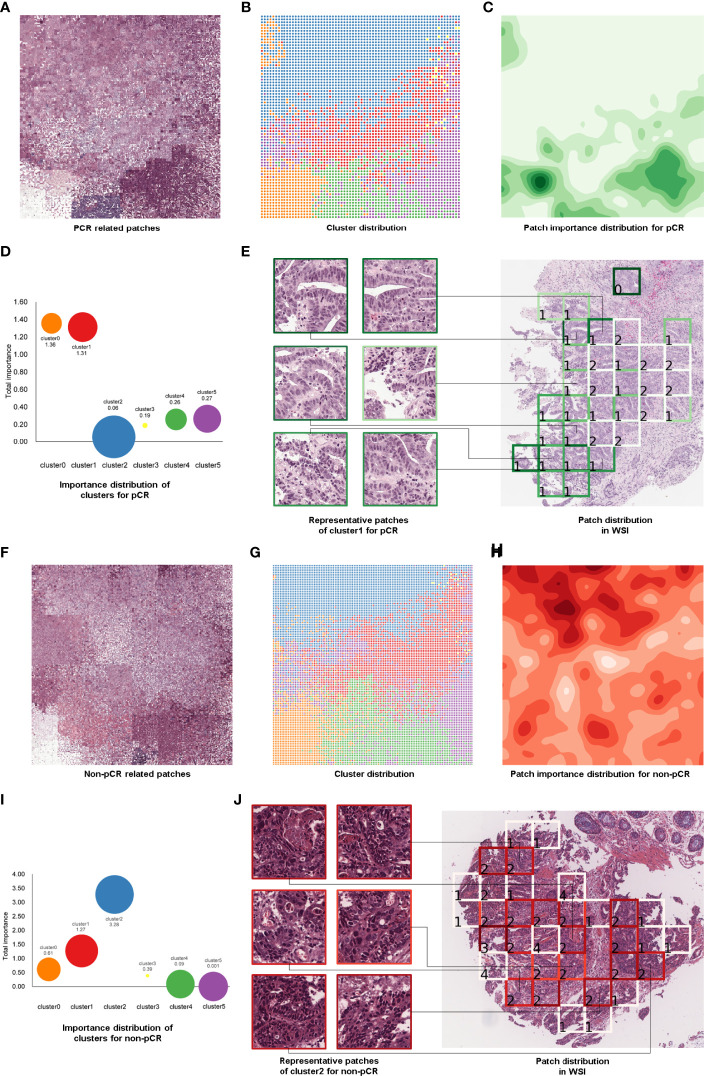
Patch-level feature interpretation in the pCR group **(A–E)** and non-pCR group **(F–J)**. Patches in the correctly predicted pCR group **(A)** and correctly predicted non-pCR group **(F)**. PatchPRs were categorized into six phenotype clusters based on t-SNE and the Raster Fairy method, and each grid represented an individual patch **(B, G)**. The importance distribution of the patches in the pCR group **(C)** and non-pCR group **(H)**. Darker colors represent the patches that played a more important role in pCR or non-pCR prediction. Demonstration of patch importance and the number of patches in each cluster; the size of the bubble represents the number of patches in the corresponding cluster **(D, I)**. Representative patches of cluster 1 **(E)** and cluster 2 **(J)** and the part of the WSI from which they were selected.

To find some important clinical insights based on the DeepPCR model, we determined which types of histological patterns were most relevant to patient TRG, and the pipeline of this process is displayed in **Figure 4**. In **Figure 4A**, each grid represented an individual patch, and the patchPRs obtained from all these patches were categorized into six phenotype clusters (**Figure 4B**), which were reduced into a two-dimensional feature space based on t-SNE and the Raster Fairy method. Here, the phenotypes could be color, edges, texture, curve and/or shape of cancer and normal tissues. To conduct an investigation into which types of phenotypes contribute the most to pCR prediction, the GradCAM method (23) was adopted to calculate the importance of patches. The importance heatmap is shown in **Figure 4C**, and darker colors indicate that the patches played a more important role in pCR prediction. We also calculated the sum of the importance values of the patches in each cluster (**Figure 4D**). It can be seen that different clusters had different predictive powers for pCR prediction, and a larger value indicated that the corresponding cluster contributed more to DeepPCR. The size of bubbles represents the number of patches in the corresponding cluster. We found that patches in clusters 0 and l played more important roles in pCR candidate prediction. Specifically, the patch importance value of cluster 1 was significantly larger than that of clusters 2, 3, 4, and 5 (*P<*0.001, *P<*0.05, *P<*0.01, and *P<*0.001, respectively). There were no significant differences between cluster 0 and cluster 1 in terms of the patch importance value (**Figure 4D**). **Figure 4E** shows the representative patches of cluster 1 and their distribution in a WSI, which also represented a special histological pattern and spatial pattern highly associated with pCR. Similarly, **Figures 4F–J** demonstrates the same patchPR visualization process but for the non-pCR group. The patch importance value of cluster 2 was significantly larger than that of clusters 0, 1, 3, 4, and 5 (*P<*0.001, *P<*0.001, *P<*0.001, *P<*0.001, and *P<*0.001, respectively) (**Figure 4I**) in non-pCR candidate prediction.

## Discussion

In the present study, we developed a novel model to predict pCR in LARC using digital pathological images. We found that the DeepPCR model could achieve a relatively high AUC-ROC score of 0.710. Multivariate logistic analysis showed that the DeepPCR model was indeed an independent factor for predicting pCR, indicating that the model could assist in treatment decision making prior to surgery for LARC.

In recent years, there has been increasing interest in digital pathology image analysis based on machine learning algorithms to assist in pathological diagnosis (24, 25). With the development of deep learning, an increasing number of studies have focused on clinical-grade detection and the prediction of outcomes. For example, Cao et al. (24) developed a pathomics-based model for microsatellite instability prediction from pathological images. Ole-Johan Skrede et al. (25) developed a deep learning-based biomarker model for colorectal cancer outcome by analyzing H&E-stained sections. The successful applications of artificial intelligence in digital pathology indicate that digital pathology images contain important information for the diagnosis and prognosis of cancer. Due to the complexity of pathological imaging, there have been few relevant studies on the prediction of neoadjuvant efficacy based on preoperative pathological biopsy with artificial intelligence. Some studies used MRI to predict neoadjuvant efficacy. For instance, Petresc et al. (8) utilized pretreatment T2-weighted radiomic features to predict LARC responders, and least absolute shrinkage and selection operator (LASSO) regression analysis was applied to derive a predicted AUC of 0.80 (95% CI: 0.58, 0.94). Although their model’s performance was better than ours, they used a small cohort of patients. In a retrospective study, Zhang et al. (10) developed a deep learning-based model for pCR prediction based on diffusion kurtosis and T2-weighted MRI, and the AUC was 0.70 (95% CI: 0.59, 0.79), which was similar to that of our proposed model. The limitation of their model was that they did not validate the model in independent external cohorts. Radiological imaging has its own limitation in distinguishing inflammatory lesions from neoplastic lesions. As the gold standard of disease diagnosis, conventional preoperative pathological biopsy is of great significance for the diagnosis and prognosis of tumors.

The discriminative power of DeepPCR model was significantly higher than that of the hematology model (*P<*0.001) and the other two patch-based models (*P<*0.001 and *P<*0.001, respectively). The number of patients in our study was larger than other reported works (8–14). Moreover, the DeepPCR model was evaluated in independent cohorts. The external validation cohort came from another center with a different sample handling procedure and using a different scanner. Although the external validation cohort was different from the primary cohort in terms of the clinicopathological characteristics, such as clinical T stage and histological grade (*P<*0.001 and *P<*0.001, respectively), the proposed model achieved similar results as those in the primary cohort, indicating its generalizability and robustness.

The proposed model leveraged an MIL-based deep learning model and showed a superior performance compared to previous patch-based learning methods. Existing patch-based approaches can be categorized into two classes based on the level of the employed annotations. For the first class, patch-wise annotations are used to train deep learning models (26–30), and strong supervision is typically performed, benefiting from the precise labeling information. Nevertheless, these methods depend on pixel-level annotations by expert pathologists, and it would be labor intensive and hard to obtain sufficient high-quality annotation data. For the second class of methods, the ground-truth labels are provided for the whole images rather than the patches (31, 32). When performing the learning process, the global image-level label of each WSI is taken as the patch-level label directly, and the final prediction is generated by combining the patch-level outputs. Although this type of method is very straightforward, there are two crucial problems. First, the cropped patches of WSIs are processed independently, and the spatial constraints of these patches are neglected. The second problem is that the patches in the same image indiscriminately share the same label and thus introduce a substantial disturbance to model training. To address these problems, several MIL-based approaches that aim to leverage the feature representations of all image patches to collaboratively predict the patient outcome have been developed (15–18, 33). Building upon these methods, our proposed model can effectively mine the dependence of feature representations at three different scales, i.e., patch-level, cluster-level, and WSI-level phenotype representations. In this patient outcome prediction task, the MIL-based learning method outperformed the patch-based learning methods. Specifically, the MIL-based methods were able to jointly consider intrapatch dependence; thus, the spatial relationships between tumor tissues (including cancer cells and surrounding stromal cells) were exploited. These tissues form the tumor microenvironment (34), and the characterization of the microenvironment plays an important role in tumor progression and the response to treatment. However, the patch-based learning methods only processed patches independently, and the spatial information among patches was neglected; thus, these methods showed poor performance. Our findings suggest that MIL-based learning models can handle the spatial information inherent in the tumor microenvironment.

Some deep learning-based studies visualized and interpreted the learned feature representations (31, 35–38), which may provide some important clinical insights. For instance, Courtiol et al. (35) identified regions that contributed to patient outcome prediction (mesothelioma classification) by visualizing various scenarios predicted by the deep learning model. They found that these regions are mostly located in the stroma and are associated with inflammation, cellular diversity and vacuolization. Campanella et al. (36) assessed the model by visualizing the features reduced in a 2D space and found that a set of top-ranked patches with probabilities close to 0.5 contained glands suspicious of being malignant. In our study, patchPRs were categorized into six phenotype clusters based on the DeepPCR model. We determined that different clusters had different predictive powers for pCR prediction. We calculated the sum of the importance values of the patches in each cluster and found that the patches in cluster 0 and cluster 1 played more important roles in pCR candidate prediction. Although we did not analyze each cluster in more detail, we proposed that some histological patterns may be associated with the predicted TRG. The novel histological pattern may be associated with the morphological features and microenvironment of the tumor.

Previous studies showed that pretreatment serum CEA levels were significantly correlated with pCR (39). In our study, the univariate logistic regression analysis showed that CEA levels significantly correlated with pCR in the primary cohort (*P=0*.033) and in the external validation cohort (*P=0*.042). However, in multivariate logistic regression analysis, this association did not persist, and only the DeepPCR model was an independent factor for predicting pCR (95% CI: 1.646, 28.743; *P*=0.008). We also conducted pCR prediction experiments based on clinical data, i.e., CEA, CA19-9, LDH, lymphocytes, and neutrophils. In the experimental studies, an AUC-ROC of 0.403 was achieved based on these nonpathological data, showing that they may not be sufficient for prognostic pCR prediction.

Although promising results and relevant clinical insights were found, there are some limitations in this study. First, this study was a retrospective study. A multicenter prospective study is needed to confirm the performance of the prediction model. Second, due to the prevalence of tumor heterogeneity, the representativeness of biopsy specimens was limited. Another limitation of this study was that deep learning has the disadvantage of its black-box nature. Although we determined some histological patterns relevant to patient TRG, the morphological features and microenvironment of each histological pattern should be further investigated.

In conclusion, our study was the first to investigate the nCRT outcome prediction problem in LARC patients using presurgical biopsy pathological images. A clinically useful prediction model was developed using deep learning. The DeepPCR model was evaluated in an independent cohort and achieved stable results. This model has the potential to guide clinicians in making nCRT choices.

## Data Availability Statement

The original contributions presented in the study are included in the article/**Supplementary Material**. Further inquiries can be directed to the corresponding authors.

## Ethics Statement

This study was approved by the Institutional Review Board of the Sixth Affiliated Hospital of Sun Yat-sen University.

## Author Contributions

JH, JPY, JHY, and YH contribute to conception and design. LF, ZL, XZ, JW, and YL contribute to acquisition of data. NZ and YF contribute to analysis and interpretation of data. NZ, YF, XL, and XF participate in drafting the article. All authors give final approval of the version to be published.

## Funding

This work was supported by the Guangdong Science and Technology Project (No. 2019B030316003 to XF), Natural Science Foundation of Guangdong Province (No. 2019A1515010901 to XF), the National Science Fund for Excellent Young Scholars (No.82122057 to XF); Guangdong Natural Science Funds for Distinguished Young Scholars (No. 2021B1515020022 to XF).

## Conflict of Interest

The authors declare that the research was conducted in the absence of any commercial or financial relationships that could be construed as a potential conflict of interest.

## Publisher’s Note

All claims expressed in this article are solely those of the authors and do not necessarily represent those of their affiliated organizations, or those of the publisher, the editors and the reviewers. Any product that may be evaluated in this article, or claim that may be made by its manufacturer, is not guaranteed or endorsed by the publisher.

## Supplementary Material

Supplementary Figure 1 Effects of the number of clusters on pCR prediction. The AUC-ROC in the test cohort was optimal when the number of clusters was set to 6.

Supplementary Figure 2 Demonstration of pCR and non-pCR patient differentiation using **(A)** patch-level, **(B)** cluster-level, **(C)** WSI-level feature representations based on the t-SNE results. With the increase in level, pCR and non-pCR candidates were easier to differentiate.
