# Supplementary figures and images for: Deep Learning Model for Predicting the Pathological Complete Response to Neoadjuvant Chemoradiotherapy of Locally Advanced Rectal Cancer
Source: Front Oncol. 2022 Jun 8;12:807264. doi: 10.3389/fonc.2022.807264 (PMC9214314; doi:10.3389/fonc.2022.807264)

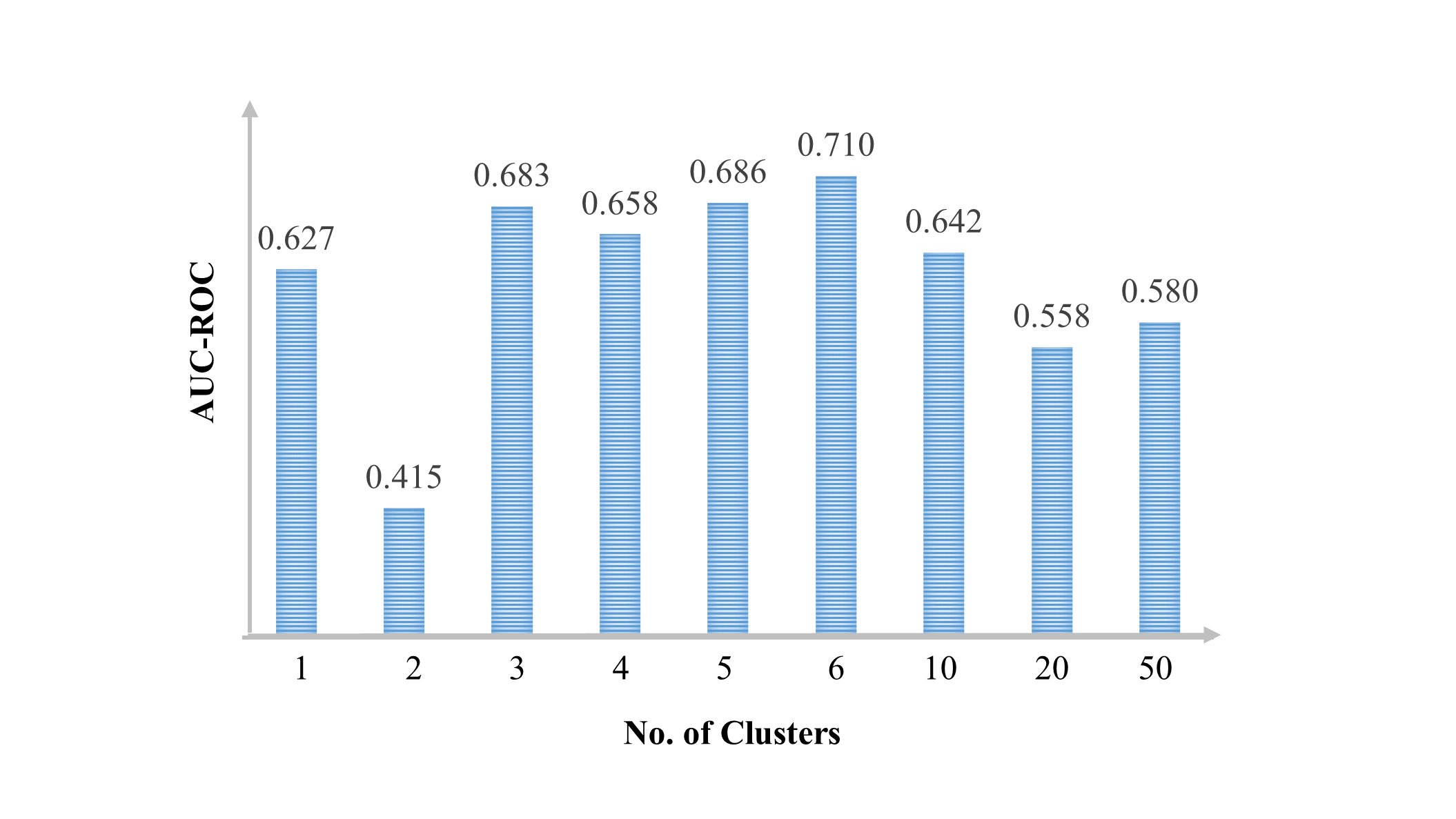

Supplement: Supplementary Figure 1 — Effects of the number of clusters on pCR prediction. The AUC-ROC in the test cohort was optimal when the number of clusters was set to 6. [file Image_1.tif]

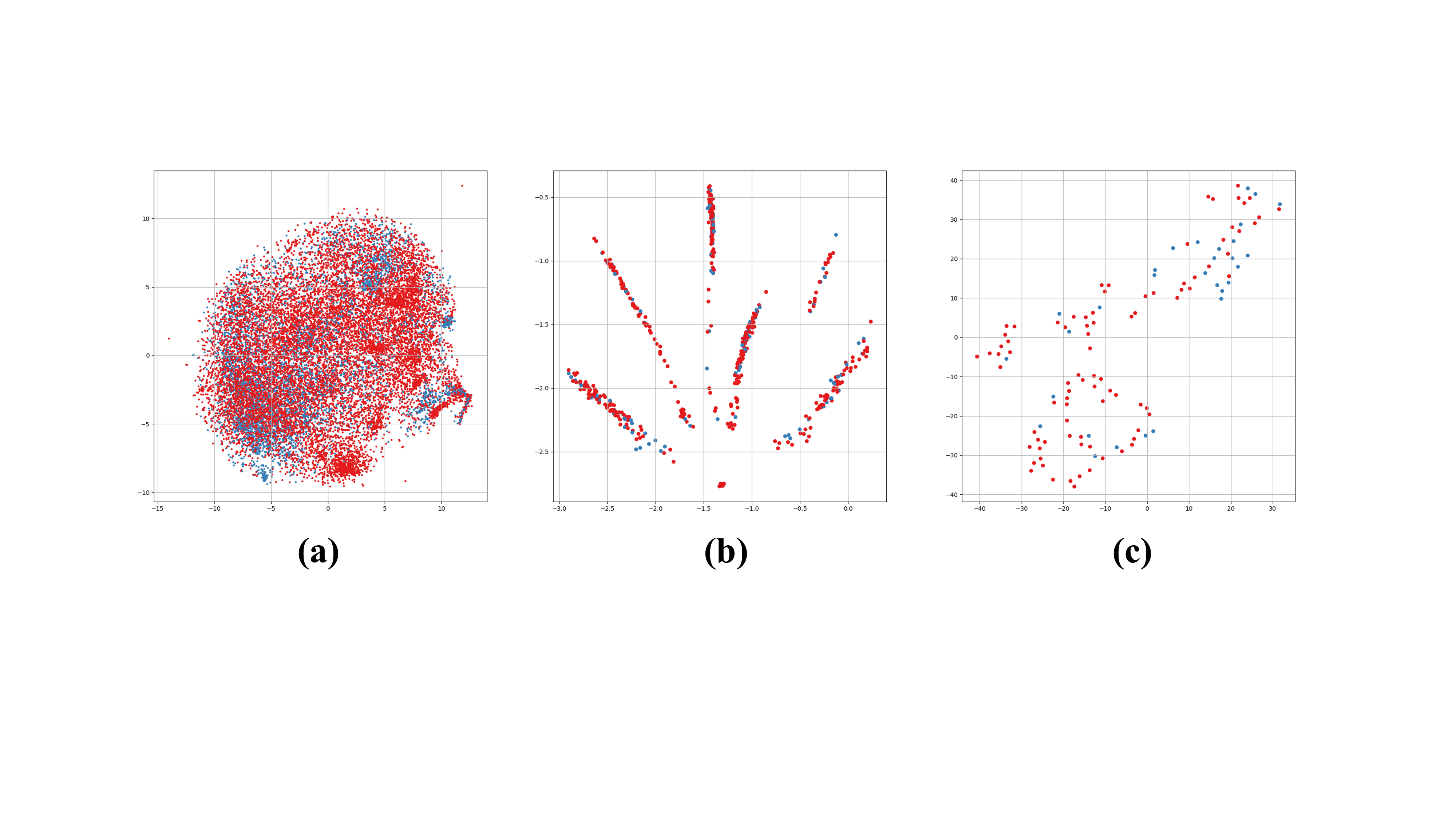

Supplement: Supplementary Figure 2 — Demonstration of pCR and non-pCR patient differentiation using (A) patch-level, (B) cluster-level, (C) WSI-level feature representations based on the t-SNE results. With the increase in level, pCR and non-pCR candidates were easier to differentiate. [file Image_2.tif]
