# Supplementary material for: Deep Learning Model for Predicting the Pathological Complete Response to Neoadjuvant Chemoradiotherapy of Locally Advanced Rectal Cancer
Source: Front Oncol. 2022 Jun 8;12:807264. doi: 10.3389/fonc.2022.807264 (PMC9214314; doi:10.3389/fonc.2022.807264)
Supplement: Supplementary file 3 [file Table_1.docx]

**Supplementary Table 1. Comparison of the clinicopathological factors in the primary and external validation cohorts.**

| Characteristics | Primary cohort  (n=783) | External  validation cohort  (n=102) | *P* value |
| --- | --- | --- | --- |
| Age, mean (SD), years | 54.33(11.79) | 56.44(10.55) | 0.063 |
| Sex, No. (%) |  |  | 0.737 |
| Female | 249(31.8) | 34(33.3) |  |
| Male | 534(68.2) | 68(66.7) |  |
| Clinical T stage |  |  |  |
| cT2 | 30(3.8) | 1(1.0) | 0.245 |
| cT3 | 511(65.3) | 33(32.4) | <0.001 |
| cT4 | 242(30.9) | 68(66.6) | <0.001 |
| Clinical N stage |  |  |  |
| cN0 | 128(16.3) | 18(17.6) | 0.777 |
| cN1 | 388(49.6) | 60(58.8) | 0.092 |
| cN2 | 267(34.1) | 24(23.6) | 0.033 |
| TNM stage |  |  | 0.777 |
| Stage II | 129(16.5) | 18(17.6) |  |
| Stage III | 654(83.5) | 84(82.4) |  |
| Histological grade |  |  |  |
| 1 | 96(12.3) | 1(1.0) | <0.001 |
| 2 | 594(75.9) | 94(92.2) | <0.001 |
| 3 | 93(11.8) | 7(6.8) | 0.182 |
| TRG |  |  | 0.717 |
| 0 | 201(25.7) | 24(23.5) |  |
| 1/2/3 | 582(74.3) | 78(76.5) |  |
| No. of patches | 121,203 | 46599 |  |
