# Supplementary material for: Deep Learning Model for Predicting the Pathological Complete Response to Neoadjuvant Chemoradiotherapy of Locally Advanced Rectal Cancer
Source: Front Oncol. 2022 Jun 8;12:807264. doi: 10.3389/fonc.2022.807264 (PMC9214314; doi:10.3389/fonc.2022.807264)
Supplement: Supplementary file 4 [file Table_2.docx]

Supplementary Table 2. Clinical characteristics of patients in the training and testing cohorts.

|  | Training set  (n=666) | |  | Testing set  (n=117) | |  |
| --- | --- | --- | --- | --- | --- | --- |
|  | PCR (%)  (n=171) | Non-PCR (%)  (n=495) | *P* value | PCR (%)  (n=30) | Non-PCR (%)  (n=87) | *P* value |
| nCRT to surgery |  |  | 0.593 |  |  | 0.395 |
| <8weeks | 80(46.8) | 219(44.2) |  | 16(53.3) | 37(42.5) |  |
| ≥8weeks | 91(53.2) | 276(55.8) |  | 14(46.7) | 50(57.5) |  |
| Tumor location (cm) |  |  |  |  |  |  |
| 0-5 | 98(57.3) | 218(44.0) | 0.003 | 16(53.3) | 41(47.1) | 0.673 |
| 5-10 | 69(40.4) | 253(51.1) | 0.012 | 14(46.7) | 39(44.8) | 0.518 |
| >10 | 4(2.3) | 24(4.9) | 0.189 | 0 | 7(8.1) | 0.188 |
| Tumor size (cm) |  |  | 0.772 |  |  | 0.247 |
| <4 | 54(31.6) | 149(30.1) |  | 6(20) | 29(33.3) |  |
| ≥4 | 117(68.4) | 346(69.9) |  | 24(80) | 58(66.7) |  |
